# Undifferentiated Intimal Sarcoma of the Visceral Aorta With Recurrent Renovisceral Ischaemia Misdiagnosed as Takayasu's Arteritis

**DOI:** 10.1016/j.ejvsvf.2025.01.002

**Published:** 2025-01-20

**Authors:** Martin Wenkel, Kirsten de Groot, Marius Fried, Achim Neufang

**Affiliations:** a Department of Cardiac and Vascular Surgery, University Medical Centre of Johannes Gutenberg University, Mainz, Germany; b 3rd Medical Department (Nephrology and Rheumatology), Sana Klinikum Offenbach, Offenbach, Germany; c Department of Haematology, University Medical Centre of Johannes Gutenberg University, Mainz, Germany

**Keywords:** Abdominal aorta, Intimal sarcoma, Vascular tumour

## Abstract

**Introduction:**

Malignant tumours of the aorta are a rare disease and often misdiagnosed as they masquerade as wall adjacent thrombus or inflammatory disease. Due to the often delayed diagnosis and the rapidly progressing illness, the outcome is very poor.

**Report:**

A 50 year old female patient who had received coeliac and mesenteric artery stenting followed by an aortomesenteric bypass after stent occlusion two years earlier was treated. After an episode of hypertensive crisis caused by high grade stenosis of the renal arteries and review of previous tissue biopsies the diagnosis of Takayasu's arteritis was established, but the results were inconclusive. When presenting with a penetrating aneurysm of the renovisceral aorta, a complete reconstruction was performed of the renovisceral aorta due to impending rupture. Pre-operative imaging incidentally showed a sarcoma of the femur which was interpreted as an unrelated entity at the time. Finally, two years after the onset of the first symptoms, the diagnosis of an undifferentiated intimal sarcoma was established after extensive histological workup. However, the patient's condition deteriorated too quickly for her to recover as she had already developed multiple distant metastases and she died within three months of surgery.

**Discussion:**

Due to its extreme rarity, this disease is not widely recognised, even among specialists. Accordingly, the patient was initially misdiagnosed and a malignant process was not considered. An accurate diagnosis at the time of the first open surgery might have presented the opportunity for radical resection of the affected aorta. Unfortunately, there are no non-invasive tools available to diagnose intimal sarcoma and, given the rapid progression of the disease, the prognosis remains poor, with a survival of only a few months.

## Highlights

• Intimal sarcomas of the aorta are exceptionally rare and are often misdiagnosed.
• They only become symptomatic when they trigger emboli or occlude large branches.
• An early diagnosis is highly unlikely and thus the outcome is almost always fatal.

## INTRODUCTION

Malignant tumours of the aorta are exceptionally rare.^1^ Remaining clinically unapparent for a long time they only become symptomatic when they trigger embolism or occlude large aortic side branches like the visceral arteries. Often misdiagnosed, the definitive diagnosis is only made after histological workup. Given the often delayed diagnosis of this rapidly progressing illness, the outcome is almost always fatal within a few months.^2^ Except biopsy, there are no diagnostic tools that allow a definitive diagnosis at an early stage. Radiological signs are too non-specific and biopsy of a radiologically conspicuous lesion is not a standard procedure.^3^ Magnetic resonance imaging (MRI) or positron emission tomography computed tomography (PET-CT) may point towards angiosarcoma but do not allow a definitive diagnosis. The case is presented of a fifty year old female who first presented with symptoms of visceral and renal ischaemia that were initially interpreted as Takayasu's arteritis before the diagnosis was established after arterial reconstruction.

## REPORT

The patient first presented in December 2020 at another institution with progressive abdominal angina and was successfully treated by stenting of the coeliac trunk and superior mesenteric artery with balloon expandable bare metal stents. She had no history of hypertension, hyperlipidaemia, or other risk factors and was a non-smoker. After freedom of symptoms for a few months, occlusion of the implanted stents with recurrent symptoms necessitated retrograde prosthetic aortomesenteric bypass. Histological examination of the aortic biopsy showed an aortitis with reactively atypical intimal cells. The patient remained free of symptoms for fifteen months, when she presented to a third hospital with a hypertensive crisis and acute kidney failure. High grade bilateral renal artery stenosis was diagnosed and primary, bilateral renal artery stenting was performed. The original aortic biopsies were reviewed, but the results remained inconclusive. As the patient complained of malaise, reduced performance for several months and had a low grade C reactive protein elevation, a provisional diagnosis of Takayasu's arteritis was made and the patient received oral prednisolone and subsequently two weekly adalimumab in order to prevent re-stenosis.

**Figure 1:**
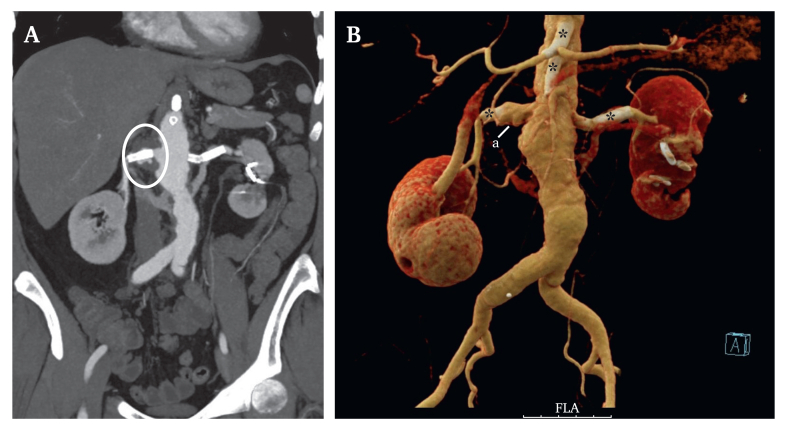
(A) Pre-operative computed tomography angiography of the aorta showing a penetrating aneurysm at the origin of the right renal artery with the dislocated stent. (B) Computed 3D reconstruction of the penetrating aneurysm. The proximal renal artery and the adjacent aortic wall are irregular shaped. (a) Renal artery; stars, occluded stents in both renal arteries, the coeliac trunk, and the superior mesenteric artery.

The then fifty year old patient again perceived post-prandial abdominal pain, weight loss, and a reocurring fever twenty four months after the initial treatment and was transferred to our hospital in December 2022 with the diagnosis of a symptomatic penetrating aneurysm of the renovisceral aorta. Furthermore, she reported lower back pain on the right side and tenderness in the upper right abdomen. Computed tomography angiography showed a penetrating aneurysm of the renovisceral aorta with partial destruction of the aortic wall (Fig. 1A). The coeliac trunk showed a high grade re-stenosis and the aortomesenteric bypass was occluded. All findings were considered as complications of the proposed Takayasu's arteritis. A pre-operative fludeoxyglucose positron emission tomography CT scan showed increased uptake of radiotracer in the renovisceral aortic segment as a sign of inflammatory vascular activity. Unexpectedly, this CT also revealed a tumour in the proximal left femur, from which a specimen was obtained. The diagnosis of an osteosarcoma was confirmed and interpreted as a primary tumour of the femur. After interdisciplinary evaluation with rheumatologists and radiologists, a plan for rapid surgical replacement of the diseased aorta was developed. Femoral resection and replacement were planned shortly after recovery from the aortic surgery.

The patient was scheduled for aortovisceral reconstruction two days later. After laparotomy ischaemic lesions were present in the caecum and an ileocecal resection was needed, which was performed by a general surgeon. In a septic operative field autologous and xenogenous reconstruction was chosen, and as a first step a retrograde mesenteric bypass using the superficial femoral vein (SFV) originating from the right common iliac artery was implanted. The right renal artery was re-implanted into the SFV graft and a great saphenous vein graft originating from the SFV graft was then used for revascularisation of the hepatic artery.

**Figure 2:**
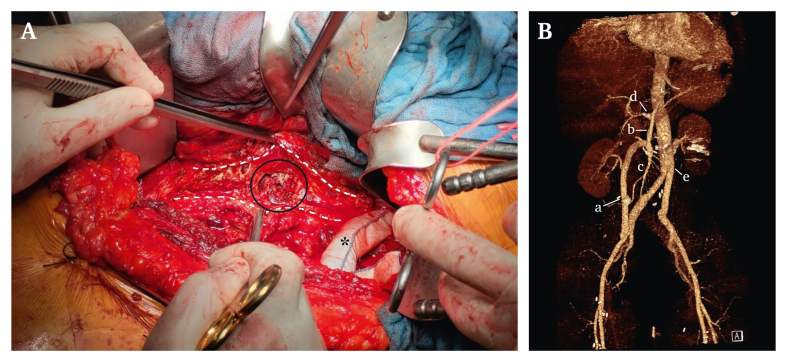
(A) Intra-operative image after longitudinal incision of the aorta (striped line). The origin of the right renal artery appeared to be completely destroyed (circle). The prepared bovine tube is already anastomosed to the proximal aorta (star). (B) Reconstruction of post-operative computed tomography angiogram showing the arterial reconstruction with aortic replacement and bypasses to the visceral and renal arteries. (a) Superficial femoral vein graft. (b) Great saphenous vein graft. (c) Superior mesenteric artery. (d) Hepatic artery. (e) Intra-operative prepared bovine pericardial Y graft.

The entire abdominal aorta was exposed by a retroperitoneal approach. Retrograde extracorporeal circulation via the femoral artery and vein was established to maintain renovisceral perfusion during supracoeliac aortic clamping. After incision of the aorta, the ostium of the right renal artery showed irregularity with a fragile and thinned out wall (Fig. 2A). The affected renovisceral segment of the aorta was resected, and an intra-operative prepared bovine pericardial Y graft with a side branch for the left renal artery implanted (Fig. 2B).

Despite initially sufficient renal function, the patient developed kidney failure two weeks post-operatively and required haemodialysis for two weeks, after which renal function recovered. The patient experienced slow convalescence from surgery but was able to be discharged from the intensive care unit on the 32nd post-operative day.

Histological workup of the resected renovisceral aorta, the ileocolic segment, and the biopsy of the femur was performed by combined analysis between the local department of pathology and a sarcoma reference centre. It showed an undifferentiated pleomorphic sarcoma of the aortic intima with positive staining for vimentin and smooth muscle actin but inconclusive for genetic markers such as CD31, CD34, and other frequent markers.

These findings were discussed at an interdisciplinary tumour board and the diagnosis of a metastatic aortic sarcoma with involvment of the left femur and the mesentery was established and the diagnosis Takayasu's arteritis was finally rejected. To avoid a pathological fracture, the affected part of the left femur was resected with prosthetic replacement eight weeks after the aortic reconstruction. Adjuvant chemotherapy with doxorubicin was planned after recovery.

Unfortunately, the patient's condition deteriorated rapidly following femoral surgery. An additional CT showed new metastases in the liver, the skin of the lower back, the abdominal wall, and in the femoral muscles of the right leg. After assessment of the new situation with a rapidly progressing illness, the patient decided against any further treatment and palliative care was initiated. The patient died 78 days after aortic surgery and twenty seven months after initial presentation with abdominal symptoms.

## DISCUSSION

**Figure 3:**
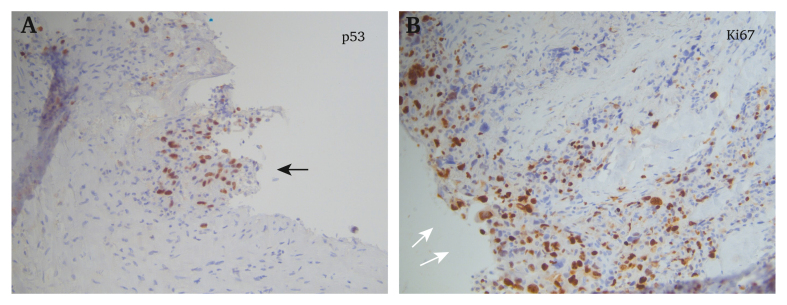
(A,B) Immunohistochemical images of the sarcoma showing aberrant p53 expression (black arrow) and high Ki67 proliferation rates (white arrow).

Sarcomas of the aorta are a very rare entity.^1^ In most cases, aortic sarcomas are initially misdiagnosed as wall adjacent thrombus, occlusive or inflammatory disease, or irregular shaped aneurysms due to their extreme rarity.^4^, ^5^, ^6^, ^7^ The definitive diagnosis can only be made after histological examination and in approximately 20% only during autopsy.^8^^,^^9^ Sarcomas can be divided into intimal sarcomas of endothelial or myofibroblastic origin and mural sarcomas of the media and adventitia.^7^ While mural angiosarcomas infiltrate surrounding tissues, intimal sarcomas grow inwards, triggering emboli or occluding major branches. In a case series of 14 patients, 39% were histologically undifferentiated with a poor prognosis. As they can be of a very heterogeneous origin, there are no specific immunohistopathological markers of intimal sarcomas but a panel including endothelial differentiation markers CD31, CD34, and von Willebrand factor may increase sensitivity (Fig. 3). However, these markers are lost when the tumour invades tissues outside the vessel wall or to metastatic sites.^10^

To date, around 220 cases have been reported. In a review of 165 cases Rusthoven *et al.* reported an alarmingly poor prognosis with a median survival between eleven and fifteen months, metastases in 44.8% at the time of diagnosis, and a five year survival of only 8.8%.^2^ In another meta-analysis Vacirca *et al.* analysed 223 cases of malignant aortic tumours. Among those, the abdominal aorta was affected in 42.6%, and the thoracic aorta in 37.7%. Risk factors for a worse outcome included hypertension, fever, back pain, low body weight, and peripheral embolisation. Once the correct diagnosis has been established, patients who receive a combination of surgery, adjuvant chemotherapy, and radiotherapy have the best outcomes with a survival rate of twelve months.^9^

Due to the extreme rarity, this disease is not widely recognised, even among specialists who are familiar with managing the differential diagnosis of intimal sarcoma, e.g. Takayasu's arteritis, which itself is a rare disease.

Accordingly, the patient was initially misdiagnosed, too. After an initial biopsy showed inflammatory cells, the patient was treated for Takayasu's arteritis, as the clinical picture of sex, patient age, and location of the arterial pathology was highly suggestive. However, at that time the histological results did not yet allow a clear diagnosis. The presence of an aortic tumour with different metastases was only assumed after the aortic specimens showed atypical and undifferentiated cells and the definitive diagnosis was made eight weeks after surgery. However, this did not alter the course of the patient as she deteriorated too quickly and died after 11 weeks. An accurate diagnosis at the time of the first open surgery might have presented the opportunity for radical resection of the aorta and the affected branches. But with only few reported cases worldwide and being the first case of an intimal sarcoma even in this experienced centre, this would have been quite unlikely.

The reported case demonstrates the importance of an interdisciplinary approach, maintaining a high level of suspicion, and thoroughly considering all available information in complex cases where symptoms and findings do not align.

## Funding

None.

## Conflicts of interest

None.
